# Redox Enzymes P4HB and PDIA3 Interact with STIM1 to Fine-Tune Its Calcium Sensitivity and Activation

**DOI:** 10.3390/ijms25147578

**Published:** 2024-07-10

**Authors:** Yangchun Du, Feifan Wang, Panpan Liu, Sisi Zheng, Jia Li, Rui Huang, Wanjie Li, Xiaoyan Zhang, Youjun Wang

**Affiliations:** 1 Beijing Key Laboratory of Gene Resource and Molecular Development, College of Life Sciences, Beijing Normal University, Beijing 100875, China; 201831200004@mail.bnu.edu.cn (Y.D.); 202221200016@mail.bnu.edu.cn (F.W.); 202231200003@mail.bnu.edu.cn (P.L.); shirley7474@163.com (S.Z.); lj2012@mail.bnu.edu.cn (J.L.); 202021200010@mail.bnu.edu.cn (R.H.); lwj@bnu.edu.cn (W.L.); 2 Key Laboratory of Cell Proliferation and Regulation Biology, Ministry of Education, College of Life Sciences, Beijing Normal University, Beijing 100875, China

**Keywords:** STIM1, STIM2, calcium affinity, P4HB, PDIA3, TXNDC5, SOCE

## Abstract

Sensing the lowering of endoplasmic reticulum (ER) calcium (Ca^2+^), STIM1 mediates a ubiquitous Ca^2+^ influx process called the store-operated Ca^2+^ entry (SOCE). Dysregulated STIM1 function or abnormal SOCE is strongly associated with autoimmune disorders, atherosclerosis, and various forms of cancers. Therefore, uncovering the molecular intricacies of post-translational modifications, such as oxidation, on STIM1 function is of paramount importance. In a recent proteomic screening, we identified three protein disulfide isomerases (PDIs)—Prolyl 4-hydroxylase subunit beta (P4HB), protein disulfide-isomerase A3 (PDIA3), and thioredoxin domain-containing protein 5 (TXNDC5)—as the ER-luminal interactors of STIM1. Here, we demonstrated that these PDIs dynamically associate with STIM1 and STIM2. The mutation of the two conserved cysteine residues of STIM1 (STIM1-2CA) decreased its Ca^2+^ affinity both in cellulo and in situ. Knockdown of PDIA3 or P4HB increased the Ca^2+^ affinity of wild-type STIM1 while showing no impact on the STIM1-2CA mutant, indicating that PDIA3 and P4HB regulate STIM1’s Ca^2+^ affinity by acting on ER-luminal cysteine residues. This modulation of STIM1’s Ca^2+^ sensitivity was further confirmed by Ca^2+^ imaging experiments, which showed that knockdown of these two PDIs does not affect STIM1-mediated SOCE upon full store depletion but leads to enhanced SOCE amplitudes upon partial store depletion. Thus, P4HB and PDIA3 dynamically modulate STIM1 activation by fine-tuning its Ca^2+^ binding affinity, adjusting the level of activated STIM1 in response to physiological cues. The coordination between STIM1-mediated Ca^2+^ signaling and redox responses reported herein may have implications for cell physiology and pathology.

## 1. Introduction

In animal cells, store-operated calcium (Ca^2+^) entry (SOCE) is an important Ca^2+^ influx process for both Ca^2+^ signaling and Ca^2+^ homeostasis [1,2,3,4]. An essential orchestrator of SOCE is the Stromal Interaction Molecule 1 (STIM1) protein, localized at the endoplasmic reticulum (ER) membrane, where it serves as a sensor of ER Ca^2+^ levels. At rest, the STIM1 cytosolic Coiled-Coil 1 (CC1) domain binds with its CRAC activation domain (CAD) or STIM1 Orai1-activating region (SOAR) to keep itself in auto-inhibition. Upon ER Ca^2+^ depletion, STIM1 molecules undergo conformational changes, releasing the SOAR domain from the CC1 region [5,6,7,8]. STIM1 proteins then oligomerize and accumulate at ER-plasma membrane (PM) junctions. Here, they interact with and activate the Orai pore-forming protein on PM, mediating Ca^2+^ influx, or SOCE [2,5,6,9]. The canonical STIM isoforms, STIM1 and STIM2, are fundamental in mediating SOCE and maintaining Ca^2+^ balance within cells. STIM proteins mediate Ca^2+^ signals in various immune, vascular, and contractile cell types, and they are implicated in a range of immune deficiency diseases and cancer [10,11,12,13]. Acquiring a comprehensive understanding of STIM activation and regulation holds promise for innovative therapeutic approaches targeting STIM-related disorders in humans.

STIM1 function can be modulated by a wide range of post-translational modifications (PTMs), such as phosphorylation [14], acylation [15,16], glycosylation [17,18], and oxidation by reactive oxygen species (ROS) [19,20]. Yet enzymes for such modifications are less well defined. Utilizing proximity labeling and imaging, we recently systemically screened and unveiled the pivotal ER-luminal interactome of STIM1 [21]. The six key ER-luminal proteins that could dynamically interact with both STIM1 and STIM2 can be divided into two groups: one related to redox reactions and the other to N-glycan processing. Earlier in vitro studies have shown that N-glycosylation decreases STIM1 Ca^2+^-binding affinity [18]. Our findings further revealed that STIM-interacting Glucosidase II (consists of PRKCSH and GANAB) is the enzyme responsible for reducing STIM1’s Ca^2+^ affinity via N-glycosylation [21].

With ROS acting as signaling molecules that modulate gene activity, transcription, and protein functions, redox reactions play crucial roles in cellular signaling pathways and affect cell processes such as differentiation, proliferation, and apoptosis [22,23,24]. Previous studies have demonstrated that redox modifications on STIM ER-luminal cysteine residues may affect its function [25,26,27,28,29,30], with STIM1-C56S mutant displaying reduced Ca^2+^-binding affinity in vitro [25,31]. Despite a report of the interaction between Endoplasmic Reticulum Protein 57 (ERp57) and STIM1 relying on the STIM1-C49-C56 residue [25], the specific redox modification enzymes affecting STIM’s Ca^2+^ affinities remain unidentified. Our recent proteomic mapping identified three redox-reacting protein disulfide isomerases (PDIs) that dynamically co-localize with STIM proteins: Prolyl 4-hydroxylase subunit beta (P4HB), ERp57 (also known as protein disulfide isomerase A3, PDIA3), and thioredoxin domain-containing protein 5 (TXNDC5) [21]. These three PDIs are reportedly involved in the formation of disulfide bonds and the folding of proteins within the ER [27,32,33]. Whether they are responsible for modulating STIM1’s Ca^2+^ affinity via redox reactions remains unclear.

In the present study, we first characterized the dynamic interactions of three oxidoreductases, namely TXNDC5, P4HB, and PDIA3, with STIM proteins. Subsequently, employing in situ Ca^2+^-titration of STIM1 via a highly dynamic FRET reporter for STIM1 activation, we unveiled that PDIA3 and P4HB could regulate STIM1’s Ca^2+^ affinity by acting on the ER-luminal conserved cysteine residues. Although this modulation of STIM1’s Ca^2+^ sensitivity does not impact its full capacity to mediate SOCE, it is likely to influence the initiation of SOCE triggered by various physiological stimuli. The elucidated molecular mechanisms herein have significantly enriched our understanding of redox modulation on Ca^2+^ signaling.

## 2. Results and Discussion

### 2.1. STIM Proteins Dynamically Interact with Three Protein Disulfide Isomerases (PDIs) (P4HB, TXNDC5, and PDIA3)

To assess the interactions between STIM proteins and the three PDI proteins (P4HB, PDIA3, and TXNDC5), we employed confocal imaging to study their dynamic co-localization, as a recent report demonstrated that direct protein–protein interactions could be identified by their dynamic co-localizations [34]. We employed two strategies to redistribute STIM proteins: triggering STIM1’s transition from a uniform to puncta ER distribution through ER-Ca^2+^ depletion [3] and reducing STIM2’s constitutive puncta via intracellular acidification [35]. Following these treatments, we evaluated the subcellular localization of these PDI proteins, both when expressed alone and when co-expressed with STIM proteins. Proteins exhibiting subcellular distribution changes solely when co-expressed with STIM were recognized as dynamic STIM interactors.

**Figure 1:**
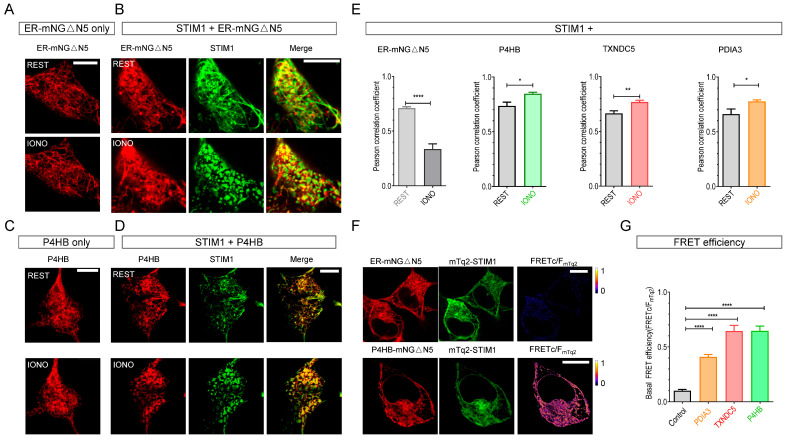
Three protein disulfide isomerases (PDIs), TXNDC5, P4HB, and PDIA3, dynamically interact with STIM1 in HEK293 cells. HEK293 cells transiently transfected with ER-localized mNeonGreen△N5 (ER-mNG△N5), P4HB-mScarlet, TXNDC5-mScarlet, or PDIA3-mScarlet alone, or co-transfected with mTurquoise2-STIM1, were examined using Airyscan super-resolution confocal imaging. Images of cells at basal condition and after 5 min ER-Ca^2+^-store depletion with 2.5 µM ionomycin (IONO) were collected from the same view field. Typical cellular images from three independent experiments are shown (more than 10 cells examined each time). STIM1 is shown in green, ER-mNG△N5 or P4HB are shown in red. Scale bar, 10 µm: (**A**) ER-mNG△N5 alone. (**B**) ER-mNG△N5 co-expressed with STIM1. (**C**) P4HB alone. (**D**) P4HB co-expressed with STIM1. (**E**) Statistics showing the extent of co-localization between STIM1 and ER-mNG△N5, P4HB, TXNDC5, or PDIA3 (n = 3, more than 30 cells examined in each group, *, *p* < 0.01; **, *p* < 0.001, Student’s *t*-test). (**F**) Typical images showing the fluorescence (left two panels) or FRET efficiency (images on the right) of cells co-expressing mTurquoise2-STIM1 and either ER-mNG△N5 (top) or P4HB-mNG△N5 (bottom) (n = 3, more than 20 cells examined each time). (**G**) Statistics showing basal FRET efficiency between mTurquoise2-STIM1 and ER-mNG△N5, PDIA3-mNG△N5, TXNDC5-mNG△N5 or P4HB-mNG△N5. (n = 3, more than 20 cells examined each time, ****, *p* < 0.0001, Student’s *t*-test).

The colocalization between STIM1 and an ER-localizing mNeonGreenΔN5 (ER-mNGΔN5) [21] served as a negative control in our experimental framework. When expressed alone, ER-mNGΔN5’s distribution remained unaltered before and after store depletion with 2.5 µM Ca^2+^ ionophore ionomycin (IONO) (Figure 1A). In cells co-expressing ER-mNGΔN5 together with STIM1, IONO induced the formation of STIM1 puncta, while the distribution of co-expressed ER-mNGΔN5 remained unaltered (Figure 1B), indicating no association between ER-mNGΔN5 and STIM1. P4HB also showed no changes in subcellular distribution upon store depletion when expressed alone (Figure 1C). However, when co-expressed with STIM1, P4HB transitioned from an even ER-like distribution to a punctate distribution after IONO treatments, showing clear colocalization with STIM1 (Figure 1D). Similarly, the other two PDIs, TXNDC5 (Figure S1A,B) and PDIA3 (Figure S1C,D), also exhibited STIM1-dependent redistribution following store depletion with IONO. We further performed Pearson correlation coefficient analysis to quantify the extent of co-localization between co-expressed proteins. The results reveal that the co-localization between ER-mNGΔN5 and STIM1 was significantly decreased upon ER depletion (Figure 1E, leftmost bar chart), while the extent of colocalization between STIM1 and all three PDIs significantly increased after ER-Ca^2+^ depletion (Figure 1E, three bar charts on the right). These results collectively demonstrate dynamic co-localizations between STIM1 and the three PDIs.

We next examined the dynamic co-localization between STIM2 and these three PDIs with confocal microscopy, using ER-mNGΔN5 as a negative control (Figures S1E–H and S2A–D). When expressed alone, ER-mNGΔN5, P4HB, TXNDC5, and PDIA3 did not exhibit redistribution following intracellular acidification (Figures S1E,G and S2A,C). In cells co-expressing ER-mNGΔN5 with STIM2, intracellular acidification greatly diminished constitutive STIM2 puncta but had no effect on the distribution of ER-mNGΔN5 (Figure S1F), suggesting no association between ER-mNGΔN5 and STIM2, with their colocalization significantly increasing upon acidification (Bar charts in Figure S1F). This phenomenon could be explained by the acidification-induced dynamic redistribution of STIM2 molecules. ER-mNGΔN5 remains uniformly distributed throughout the ER, regardless of ER luminal pH. However, under resting conditions, constitutively active STIM2 proteins are primarily localized at the ER-PM junctions, resulting in minimal colocalization with ER-mNGΔN5 within the bulk of the ER. Upon acidification, these constitutive punctate STIM2 transition to an auto-inhibitory state and distribute uniformly throughout the ER, thereby increasing their colocalization with ER-mNGΔN5 within the bulk of the ER. In contrast to ER-mNGΔN5, P4HB (Figure S1H), TXNDC5 (Figure S2B) and PDIA3 (Figure S2D) also exhibited STIM2-dependent transition in cellular distribution, with their colocalization significantly diminishing upon acidification (Bar charts in Figures S1H and S2B,D). Together, these results similarly reveal dynamic co-localizations between the three PDIs and STIM2.

To quantify the associations between STIM1 and P4HB, PDIA3, or TXNDC5 at the nanometer scale, we evaluated the basal FRET signals between these PDI proteins and co-expressed STIM molecules, with the non-STIM-interacting ER-mNG△N5 serving as a negative control. Despite exhibiting good colocalization with both STIM1 and STIM2 (Figure 1E, leftmost panel; Figure S1F, bar chart), ER-mNG△N5 showed minimal basal FRET signals with STIM proteins (Top panels in Figure 1F and Figure S2E). In contrast, the resting FRET signals between P4HB and STIM molecules were significantly higher (Bottom panels in Figure 1F and Figure S2E). Similarly, the basal FRET signals between STIM proteins and PDIA3, or TXNDC5 were also significantly higher than negative control (Figure 1G and Figure S2F). These results thus strongly suggest that P4HB, TXNDC5, or PDIA3 may physically associate with both STIM1 and STIM2 (Figure 1F–G and Figure S2E,F).

PDIA3 has been shown to modulate STIM1-mediated SOCE [25]. Interestingly, among the three STIM-interacting PDIs, PDIAs showed the lowest basal FRET signal with STIM proteins (Figure 1G and Figure S2F). This suggests that the other protein disulfide isomerases may likely affect SOCE, possibly via their redox modifications on STIM proteins.

### 2.2. STIM Mutants Lacking Disulfide-Bond-Forming Ability Exhibited Lower Ca^2+^-Binding Affinity

**Figure 2:**
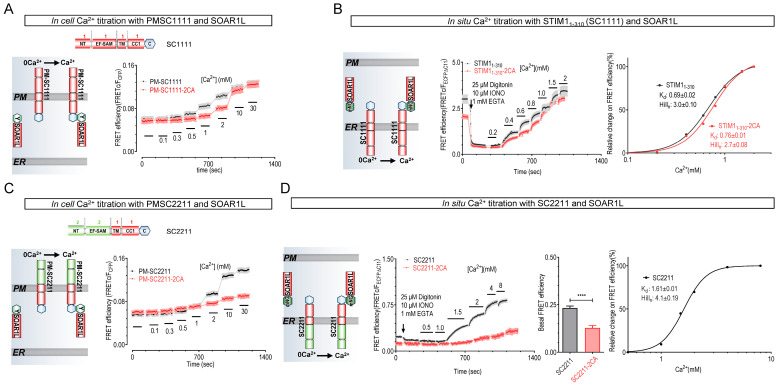
The effect of STIM1 C49A-C56A (2CA) or STIM2 C140A-C147A (2CA) mutation on their Ca^2+^-binding affinities in HeLa STIM1-STIM2 double knockout (HeLa SK) cells. Cartoons on the left are depictions of the types and locations of SC constructs used. (**A**) In-cell Ca^2+^ titration responses represented by FRET signals between co-expressed YFP-SOAR1L and PM localized STIM1_1-CC1_ constructs, wild-type (WT) PM-SC1111-CFP, or PM-SC1111-2CA-CFP. Right, typical traces. (n = 3, more than 50 cells per measurement). (**B**) in situ Ca^2+^ titration curves shown by FRET signals between mNG△N5-SOAR1L and ER-localized STIM1_1-CC1_ constructs, WT STIM1_1–310_-CFP△C11, or STIM1_1–310_-2CA-CFP△C11. Middle, typical FRET response; right, Ca^2+^ titration curves. (n = 3, more than 50 cells per measurement). (**C**) In-cell Ca^2+^ titration responses represented by FRET signals between co-expressed YFP-SOAR1L and PM localized STIM2_1-CC1_ constructs, WT PM-SC2211-CFP or PM-SC2211-2CA-CFP. Right, typical traces. (n = 3, more than 50 cells per measurement). (**D**) in situ Ca^2+^ titration curves shown by FRET signals between mNG△N5-SOAR1L and ER-localized STIM2_1-CC1_ constructs, WT SC2211-CFP△C11, or SC2211-2CA-CFP△C11. Middle left, typical RERT response; middle right, statistics of basal FRET efficiency; rightmost, Ca^2+^ titration curves. (n = 3, more than 50 cells per measurement, ****, *p* < 0.0001, Student’s *t*-test).

To investigate whether redox modifications of two conserved cysteine residues (2C) in the ER-luminal region of STIM molecules (STIM1-C49-C56 or STIM2-C140-C147) affect their function, we utilized a FRET assay recently developed by us to examine the effects of mutating the 2C residues into Alanine (2CA) on their ability to bind Ca^2+^ [36]. In the assay, we used engineered, PM-localized STIM constructs to expose the luminal Ca^2+^-binding EF-SAM domain of STIM to the extracellular space (PM-SC1111, Figure 2A), allowing precise manipulation of Ca^2+^ levels in the vicinity of the extracellular EF-SAM [36]. We previously demonstrated their successful localization to the PM and their ability to sense fluctuations in extracellular Ca^2+^ levels [36]. Extracellular Ca^2+^-induced FRET responses mediated by cytosolic PM-SC and YFP-SOARL (STIM1_343–491_), a longer version of SOAR, could be used to faithfully deduce the in cellulo Ca^2+^-binding affinities of these STIM constructs. To avoid artifacts induced by endogenous STIM1 or STIM2 molecules and the filling status of the ER Ca^2+^ stores, the FRET experiments were performed in HeLa STIM1 and STIM2 double knockout (SK) cells.

The in-cell Ca^2+^ titration results show that the PM-SC1111-C49A-C56A (PM-SC1111-2CA) mutant exhibited lower Ca^2+^-binding ability (Figure 2A). We subsequently measured the in situ K_d_ value of STIM1 using an improved FRET tool with significantly larger dynamics. In this new tool, STIM1_1–310_-ECFP△C11 (SC1111-ECFP△C11) and mNG△N5-SOAR1L serve as readouts for STIM1 activation [21]. The results show that the in situ Ca^2+^ affinity of STIM1 is 0.69 ± 0.02 mM, with a Hill number of 2.9 ± 0.1. This Hill coefficient is similar to our previous observation [36], indicating a significant positive synergistic effect during the activation of truncated STIM1 dimers, while the Hill number measured with a full-length STIM1 is significantly higher (Hill_n_ = 9.7) [37], likely reflecting STIM1’s oligomerization facilitated by PM binding via the C-terminal K-rich region. By lacking the K-rich region, our tool is suitable for dissecting the initial stages of STIM1 activation. Given that both the in cellulo data and the in situ results were obtained using the same FRET readout in the same type of cells, the observed differences in Ca^2+^ binding clearly suggest the presence of potential additional modulators or post-translational modifications (PTMs) of STIM within the ER lumen. These findings are consistent with those previously reported by our laboratory [36]. The measured K_d_ value of STIM1_1–310_-2CA (0.78 ± 0.01 mM) was significantly higher than wild-type (WT) STIM1_1–310_ (Figure 2B). Meanwhile, our Western blot analysis results indicate that C49-C56 residues in STIM1 form disulfide bonds (Figure S3A). These results suggest that the breaking of disulfide bonds decreases STIM1’s Ca^2+^ affinity. This aligns with a previous in vitro report showing that the STIM1-23-213 fragment with Cys49Ser-Cys56Ser mutation exhibited a higher K_d_ value compared with WT STIM1-23-213 [31].

We then investigated the effects of the 2CA mutation on STIM2’s K_d_ value for Ca^2+^ using similar in cellulo and in situ assays. Since the binding of STIM2 SOAR with its CC1 region is weaker compared with those of STIM1, we utilized STIM1 chimeric constructs in which the luminal Ca^2+^-binding EF-SAM region was swapped with that of STIM2 (SC2211) to better report the activation of STIM2. Unfortunately, the 2CA mutant of the PM version of SC2211 (PM-SC2211-C140A-C147A, or PM-SC2211-2CA) showed impaired plasma membrane localization and Ca^2+^ responses, limiting further investigation (Figure 2C). We then compared the in situ Ca^2+^ affinity of STIM2 EF-SAM and its corresponding 2CA mutant (SC2211-C140A-C147A, or SC2211-2CA) (Figure 2D). Similar to in cellulo observation, SC2211-2CA exhibited greatly diminished Ca^2+^ responses, hindering accurate estimation of its Ca^2+^ affinity (Figure 2D, red trace, middle left panel). Nevertheless, SC2211-2CA exhibited a notably lower basal FRET signal with SOAR1L compared with the wild type, indicating a reduced Ca^2+^ affinity (Figure 2D, bar chart).

Of note, the in situ K_d_ value of STIM2 is 1.61 ± 0.01 mM (Figure 2D, rightmost panel), much higher than previous in situ measurements [38,39]. Since our high signal-to-noise assay utilized direct manipulation of ER Ca^2+^ levels, avoiding artifacts from inaccurate estimation using ER Ca^2+^ indicators with insufficiently low affinity, the value reported herein likely provides a more accurate estimation of STIM2’s affinity. The basal ER Ca^2+^ level in HEK293 cells, estimated using our newly developed highly sensitive ER Ca^2+^ indicator [40], TuNer-s, is 1.44 mM. Therefore, based on calculations using the Hill equation, approximately 75% of STIM2 molecules exist in Ca^2+^-free, active state, elucidating its well-documented constitutively active nature [3]. Furthermore, the observed Ca^2+^-binding ability of STIM2 is considerably lower than that of STIM1 (Figure 2D, rightmost panel, and Figure 2B), which is consistent with previous findings from our group and others [36,38,39].

Collectively, these in cellulo and in situ results clearly demonstrate that 2CA mutations reduce Ca^2+^ affinities of STIM proteins. STIM2-2CA mutants exhibited diminished dynamics, indicating that redox modifications within its luminal region may impair its activation, thereby hindering further dissection. Ca^2+^ imaging results show that 2CA mutation has no significant effect on STIM2-mediated constitutive Ca^2+^ entry, indicated by GEM-GECO1 [41] (STIM2: 7.5 ± 0.30, STIM2-2CA: 7.3 ± 0.35), indicating that redox modifications may have minimal impact on STIM2-mediated Ca^2+^ responses. Consequently, our attention focused on STIM1 for the remaining study.

### 2.3. Knocking down P4HB or PDIA3 Reduce the Ca^2+^ Affinity of STIM1

**Figure 3:**
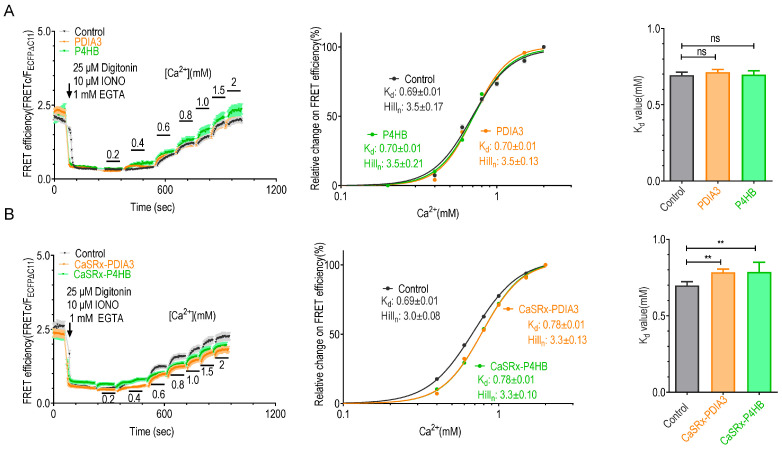
The effects on in situ STIM1’s Ca^2+^ affinity by manipulating the expression of STIM1-interacting protein disulfide isomerases in HeLa SK cells. Ca^2+^-binding behavior was measured by FRET responses between co-expressed mNG△N5-SOAR1L and STIM1_1–310_-CFP△C11. Left, typical traces of FRET efficiency; middle, Ca^2+^ titration curves; right, statistics. (**A**) Effects of PDIA3 or P4HB co-expression (n = 3, more than 30 cells per measurement, ns, not significant, Student’s *t*-test). (**B**) Effects of PDIA3 or P4HB knockdown. (n = 5, more than 30 cells per measurement, **, *p* < 0.001, paired Student’s *t*-test).

We next set out to explore the impact of the three STIM-interacting PDIs on STIM1’s Ca^2+^-binding behavior. We first examined the effects of P4HB, PDIA3, or TXNDC5 overexpression on Ca^2+^-induced FRET responses between STIM1_1–310_ (SC1111) and SOAR1L. We performed Western blot analysis to quantify and compare the levels of overexpressed PDIA3, P4HB, or TXNDC5 proteins relative to their endogenous counterparts (Figure S3B). Our results demonstrate that the overexpression of PDIA3, P4HB, or TXNDC5 did not significantly change the levels of their respective endogenous counterparts (Figure S3C). Furthermore, the total levels of these aforementioned proteins in overexpressing cells were markedly higher than those in blank control cells (Figure S3D). Interestingly, the overexpression of P4HB, PDIA3 (Figure 3A), or TXNDC5 (Figure S4A) did not significantly modify the K_d_ values of STIM1. It is likely that endogenous levels of these proteins are sufficient to make necessary redox modifications on STIM1. We thus proceeded to investigate the effects of knocking down these proteins using CasRx technology [42]. The efficiency of the knockdown was assessed with quantitative RT-PCR, revealing a significant decrease in their mRNA levels (Figure S4B). This was further confirmed by Western blot analysis, demonstrating a notable reduction in the expression of these proteins (Figure S4C,D). Interestingly, knocking down TXNDC5 did not affect the K_d_ value of STIM1 (Figure S4E). This might be attributed to the less sufficient knockdown efficiency of TXNDC5 (Figure S4B–D, red) or the existence of alternative regulatory processes that compensate for TXNDC5 function within cells. In contrast, in cells with PDIA3 or P4HB knockdown, the K_d_ values of STIM1 significantly increased compared with those in control cells (Control: 0.69 ± 0.01 mM; CasRx-PDIA3: 0.78 ± 0.01 mM; CasRx-P4HB: 0.78 ± 0.01 mM) (Figure 3B). These findings highlight the critical roles of both PDIA3 and P4HB in regulating STIM1’s Ca^2+^ affinity.

**Figure 4:**
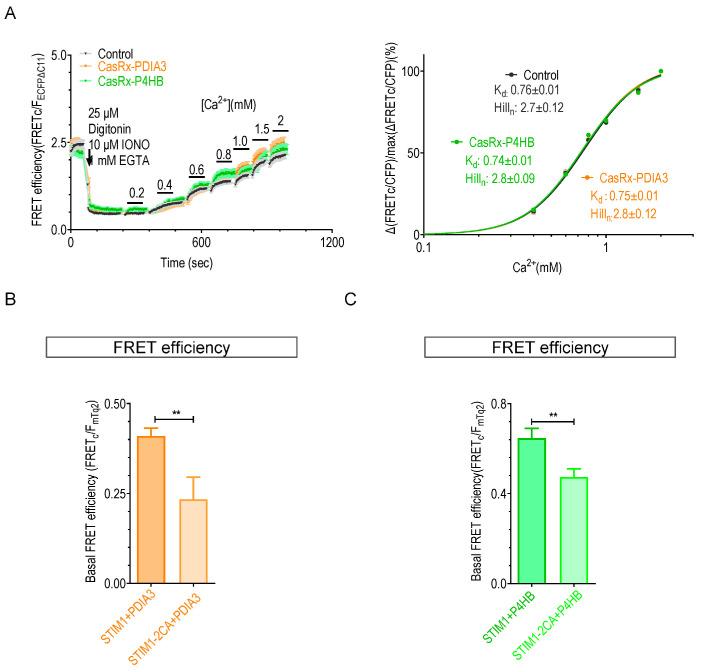
Effects of STIM1-2CA mutation on the abilities of PDIA3 or P4HB to associate with STIM1 or modulate STIM1’s Ca^2+^-binding affinity: (**A**) In situ Ca^2+^ titration curves regarding FRET responses between mNGΔN5-SOAR1L and STIM1_1–310_-2CA-CFPΔC11 constructs in cells transfected with control CasRx, CasRx-PDIA3, or CasRx-P4HB. The left panel illustrates traces of Ca^2+^ response; the right panel presents Ca^2+^ titration curves (n = 3, Student’s *t*-test, with more than 30 cells examined per measurement). (**B**) Statistics showing basal FRET efficiency between PDIA3-mNG△N5 and mTurquoise2-STIM1 or mTurquoise2-STIM1-2CA (n = 3, more than 20 cells examined each time, **, *p* < 0.009, Student’s *t*-test). (**C**) Statistics showing basal FRET efficiency between P4HB-mNG△N5 and mTurquoise2-STIM1 or mTurquoise2-STIM1-2CA (n = 3, more than 20 cells examined each time, **, *p* < 0.003, Student’s *t*-test).

To assess whether these regulatory effects are related to redox reactions on STIM1-C49-C56 residues, we examined the impact of knocking down PDIA3 or P4HB on the K_d_ values of STIM1-2CA mutants lacking the ability to form disulfide bonds. The results show that knocking down PDIA3 or P4HB did not alter the K_d_ value of STIM1-2CA (Figure 4A), indicating that PDIA3 or P4HB alter the Ca^2+^-binding behavior of STIM1 by their actions on STIM1-C49-C56 residues. Furthermore, the resting FRET signals between STIM1-2CA and PDIA3 or P4HB were significantly reduced compared with those detected with wild-type STIM1 (Figure 4B,C), indicating that these two conserved residues are crucial for the interactions of PDIA3 or P4HB with STIM1. Interestingly, the 2CA mutation showed no significant effect on the basal FRET efficiency between STIM1 and TXNDC5 (STIM1 + TXNDC5: 0.64 ± 0.05; STIM1-2CA + TXNDC5: 0.61 ± 0.12; ns, not significant, Student’s *t*-test). Thus, C49-C56 residues of STIM1 appear nonessential for its binding with TXNDC5, indicating a different interaction mode between TXNDC5 and STIM1. Consistent with prior findings [25], knockdown of PDIA3 or P4HB did not significantly alter the disulfide bond formation of STIM1. This suggests the presence of additional redox enzymes compensating for the function of PDIA3 or P4HB (Figure S4F). These findings collectively suggest that the interaction of PDIA3 and P4HB with the cysteine residues of STIM1 is required to regulate its Ca^2+^ sensitivity.

### 2.4. The Knockdown of P4HB or PDIA3 Promotes STIM1 Activation and SOCE

**Figure 5:**
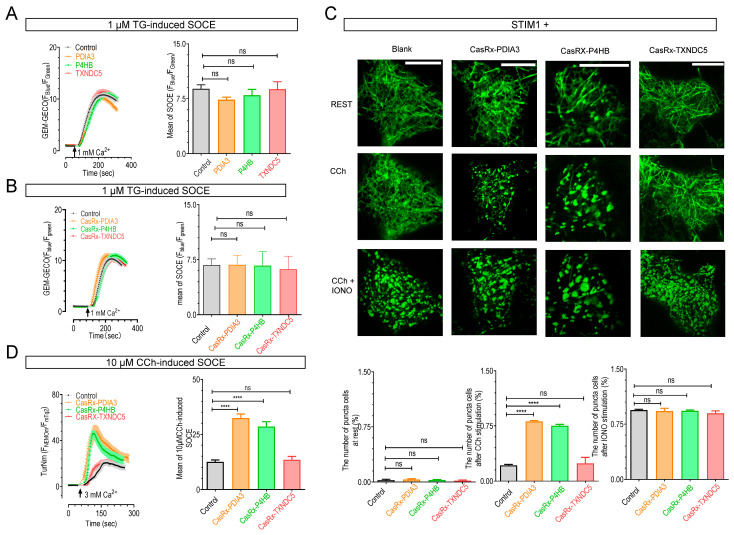
Effects of PDIA3, P4HB, or TXNDC5 on STIM1 activation and STIM1-mediated SOCE responses in HEK293 cells: (**A**,**B**) SOCE responses in HEK293 cells stably expressing a cytosolic Ca^2+^ indicator, GEM-GECO1 (GEM-GECO1 cells), transiently transfected with mScarlet tagged PDIA3, P4HB, or TXNDC5 (**A**), or corresponding sgRNAs (**B**). Prior to recordings, cells were incubated in nominally Ca^2+^-free solutions containing 1 μM thapsigargin (TG) for 10 min. An amount of 1 μM TG was present throughout recordings. (**A**) Effects of overexpression. Black, control; green, mScarlet-P4HB; orange, PDIA3-mScarlet; red, TXNDC5-mScarlet (n = 3, more than 50 cells per measurement, ns, not significant, Student’s *t*-test). (**B**) Effects of knockdown. Black, control; green, CasRx-P4HB; orange, CasRx-PDIA3; red, CasRx-TXNDC5. (n = 3, more than 50 cells per measurement, ns, not significant, Student’s *t*-test). (**C**) Representative images showing the distribution of mTurquoise2-STIM1 in cells co-transfected with mTurquoise2-STIM1 and various sgRNAs. Images were taken at rest, 5 min after stimulation with 10 µM Carbachol (CCh), or 5 min after subsequent addition of 2.5 µM IONO. Bottom: statistical analysis (n = 3, more than 20 cells per measurement, ****, *p* < 0.0001; ns, not significant, Student’s *t*-test, scale bar, 10 µm). (**D**) 10 μM CCh-induced SOCE in HEK293 cells stably expressing a highly sensitive cytosolic Ca^2+^ indicator, TurNm [40] (TurNm cells), transiently transfected with empty vector, CasRx-P4HB, CasRx-TXNDC5, or CasRx-PDIA3. Prior to recordings, cells were incubated in nominally Ca^2+^-free solutions containing 10 μM CCh for 5 min. A total of 10 μM CCh was present throughout recordings (n = 3, more than 50 cells per measurement, ****, *p* < 0.0001, Student’s *t*-test).

We further investigated the functional consequences of these interactions by firstly examining the effects of PDIA3 or P4HB on STIM1-mediated SOCE responses. HEK293 cells stably expressing a cytosolic Ca^2+^ indicator, GEM-GECO1 [41] (GEM-GECO1 cells), were transiently transfected with PDIA3-mScarlet or P4HB-mScarlet. After passive depletion of the ER Ca^2+^ store with 1 μM thapsigargin (TG), an inhibitor of ER Ca^2+^ pump, 1 mM extracellular Ca^2+^ was added to an extracellular bath to allow Ca^2+^ influxes via SOCE. Compared with blank controls, GEM-GECO1 cells expressing PDIA3, P4HB, or TXNDC5 exhibited GEM-GECO1 responses of similar amplitudes (Figure 5A), indicating no significant alteration in SOCE responses. Similarly, knocking down PDIA3, P4HB, or TXNDC5 still did not affect the amplitudes of SOCE indicated by GEM-GECO1 responses (Figure 5B). These results demonstrate that PDIA3, P4HB, or TXNDC5 does not affect SOCE signals activated by maximal ER Ca^2+^ store depletion. Unlike a previous report that found PDIA3 inhibits SOCE in mouse embryonic fibroblasts (MEFs) [25], our results align with reports in HEK293 cells, where the STIM1-C49S-C56S mutant mediates SOCE with amplitudes similar to WT STIM1 [27,30]. It appears that the PDIA3’s effect on SOCE may be cell-type-specific. Nevertheless, our findings are consistent with their ability to modify STIM1’s K_d_ value for Ca^2+^. Thus, upon complete store depletion with TG, all STIM1 molecules, regardless of whether their affinities are altered by PDIs, will be all activated, resulting in SOCE with similar amplitudes in HEK 293 cells.

Subsequently, we investigated the effects of knocking down (KD) these PDIs on STIM1-dependent signaling triggered by submaximal stimulation mimicking physiological conditions. We envisioned that PDIA3 or P4HB KD would reduce STIM1’s K_d_ value for Ca^2+^, making them more sensitive to partial ER Ca^2+^ store depletion. We first assessed the formation of STIM1 puncta, indicative of STIM1 activation [3], triggered by submaximal activation of muscarinic acetylcholinergic receptors with 10 μM carbachol (CCh). Following knockdown of TXNDC5, there was no significant difference in the proportion of cells forming STIM1 puncta in response to 10 μM CCh compared with blank controls. Interestingly, a larger proportion of cells with PDIA3 or P4HB KD exhibited STIM1 puncta following CCh stimulation compared with blank control cells (Figure 5C), suggesting heightened STIM1 activation in PDIA3 or P4HB KD cells but not in TXNDC5 KD cells. We next examined the effects of PDIs KD on STIM1-mediated SOCE induced by 10 µM CCh. However, SOCE amplitudes triggered by partial store depletion with 10 µM CCh were quite small. Although within GEM-GECO1’s linear range (Kd~340 nM) [41], the response was too small for accurate quantification. Therefore, we used a higher Ca^2+^ concentration (3 mM) along with a more sensitive Ca^2+^ indicator TurNm [40] to ensure enhanced detection of SOCE responses (Figure S4G). The knockdown of TXNDC5 didn’t affect the magnitude of 10 μM CCh-induced SOCE (Figure 5D), which aligns with the effect on the K_d_ value of STIM1 observed upon knocking down TXNDC5. Interestingly, the results reveal a significantly larger CCh-induced SOCE in cells with PDIA3 or P4HB KD (Figure 5D). These findings collectively indicate that the knockdown of PDIA3 or P4HB renders STIM1 more readily activated, potentially resulting in increased SOCE in response to physiological stimuli.

## 3. Materials and Methods

### 3.1. Plasmids Construction

mNeonGreen [43] and mScarlet [44] plasmids were kind gifts from Dr. Chen Liangyi, Peking University. The coding sequences (CDS) of P4HB, TXNDC5, and PDIA3 were gifts from Professor Qian Zhaohui. To generate an ER-localized ER-mNeonGreenΔN5, we first synthesized a forward primer containing CDS of the signal peptide from calreticulin and a reverse primer that included the CDS of ER retention sequence KDEL. Subsequently, PCR amplification was performed using the mNeonGreen template, and the product was subcloned into pcDNA3.1(+) using a multiple-fragment homologous recombination kit (Catalog Number: C113, Vazyme Biotech, Nanjing, China). To generate the mScarlet-tagged P4HB construct, the corresponding sequences of mScarlet and P4HB were PCR-amplified, and subsequently subcloned into the pcDNA3.1(+) backbone using the same kit. A similar method was used to generate TXNDC5-mScarlet, TXNDC5-mNG△N5, P4HB-mNG△N5, PDIA3-mNG△N5, and PDIA3-mScarlet. STIM1_1–310_ amplified from wild-type STIM1 [36] and ECFP△C11 were inserted into the pcDNA3.1(+) backbone to generate STIM1_1–310_-ECFP△C11 employing the same kit. A similar method was used to generate mNG△N5-SOAR1L. To construct P4HB, TXNDC5, or PDIA3 knockdown plasmid, we design the gRNA targeting the CDS of P4HB: 5′-GTGTGGTCACTGCAAACAGTTG-3′, TXNDC5: 5′-CGAAACTGTCAAGATTGGCAAG-3′ or PDIA3: 5′-CCAACACTAACACCTGTAATAA-3′. The gRNA oligos were annealed and subsequently cloned into the pAK_DR30_EF1a_CasRx_Puro vector [42] (Addgene plasmid #134842) linearized by BsmBI (New England Biolabs, Ipswich, MA, USA) with T4 ligase (New England Biolabs, Ipswich, MA, USA). All plasmids were confirmed by sequencing.

### 3.2. Gene Knockdown by CasRx

Knockdown of PDIA3, P4HB, or TXNDC5 in HeLa STIM1 and STIM2 double knockout (SK) cells was achieved via the CasRx system [42]. Cells were transfected with corresponding CasRx plasmids via electroporation, and the transfected cells were used for further experiments after 48 h of transfection. The efficiency of CasRx transfection was confirmed by qPCR and Western blot analysis.

### 3.3. Total RNA Isolation and Quantitative Real-Time Polymerase Chain Reaction (qPCR) Analysis

Total RNA was extracted from cells using TRIzol, Waltham, MA, USA, and reverse transcription was performed with PrimeScript™ RT Master Mix (Takara, cat. no. RR036A, Kusatsu, Japan) following the manufacturer’s instructions. The cDNA product was used as a template for qPCR run and mixed with primers (Table 1) and SYBR Green PCR Master Mix (GenStar Biosolutions, cat. no. A314, Beijing, China). qPCR reaction was performed on QuantStudio™ 6 Flex Real-Time PCR System (Applied Biosystems, Foster City, CA, USA). Relative mRNA levels were calculated using the Comparative Ct (△△CT) method. Gene expression levels were normalized to those of human GAPDH [45].

### 3.4. Cell Culture and Transfection

HEK293 and HeLa cells (ATCC, catalog nos. CRL-1573 and CL-0101, respectively) were cultured in DMEM (Cytiva, Marlborough, MA, USA) supplemented with 10% FBS (ExCell, Shanghai, China) and 1% penicillin and streptomycin at 37 °C with 5% CO_2_ [36,46]. Transfections were performed by electroporation using the Gene Pulser Xcell system (Bio-Rad, Hercules, CA, USA) in 4 mm cuvettes and OPTI-MEM medium. For HEK293 cells, a voltage step pulse (180 V, 25 ms, in 0.4 mL of the medium) was used; for HeLa cells, an exponential pulse (260 V, 525 µF, in 0.5 mL medium) was used. Transfected cells were seeded on round coverslips, first cultured in serum-free OPTI-MEM for 40 min, then in regular DMEM medium containing 10% FBS and 1% P/S for 24 h.

To establish stable cells stably expressing GEM-GECO1 [41], the Ca^2+^ indicator GEM-GECO1 was transfected into HEK293 cells. After selection with 2 μg/mL puromycin for 5–7 days, the cells were then diluted to single clones and expanded in culture. Healthy clones with high expression and normal Ca^2+^ responses were selected for usage.

### 3.5. Ca^2+^ Imaging in Living Cells

All Ca^2+^ imaging experiments were performed as previously described [36,47]. Cells seeded on the round coverslips were transferred to an imaging chamber and incubated in imaging buffer containing 107 mM NaCl, 7.2 mM KCl, 1.2 mM MgCl_2_, 11.5 mM glucose, and 20 mM HEPES-NaOH (pH 7.2). Time-lapse fluorescence images were acquired every two seconds with a ZEISS observer Z1 imaging system controlled by the SlideBook software v.6.0.23 (Intelligent Imaging Innovations, Denver, CO, USA) [48]. All imaging experiments were performed at room temperature. Ca^2+^ signals were indicated by ratiometric-GECIs: GEM-GECO1, TurNm [40]. Filters for GEM-GECO1 (325–402 nm _Ex_, 414–480 nm and 526–557 nm _Em_), and for TurNm (NEMO_m_: 500 ± 10 nm _Ex_; 535 ± 15 nm _Em_; sfTq2^OX^: 438 ± 12 nm _Ex_, 470 ± 12 nm _Em_) were used. Mean fluorescence readings from regions of interest were exported and processed with Matlab 2023b (The MathWorks, Natick, MA, USA) to calculate the relative ratio of F_blue_/F_green_ or F_NEMOm_/F_sfTq2OX_ indicating the changes in cytosolic Ca^2+^ concentration, then plotted with Prism 9.5.1. Traces shown are representative of at least three independent repeats, each including 30–60 single cells.

### 3.6. Förster Resonance Energy Transfer (FRET) Imaging and Measurements of Ca^2+^ Affinity of STIM1

The above-mentioned ZEISS observer Z1 system for Ca^2+^ imaging was used for the FRET imaging, with calibrations and offline analysis performed as previously described [49,50,51]. In this study, three pairs of fluorescent proteins, CFP and YFP, ECFP△C11 and mNG△N5, and mTurquoise2 and mNG△N5, were used. Fluorescence (F) of CFP/ECFP△C11/mTurquoise2 (438 ± 12 nm _Ex_/483 ± 16 nm _Em_), YFP/mTurquoise2 (510 ± 5 nm _Ex_/542 ± 13.5 nm _Em_), and FRET _raw_ (438 ± 12 nm _Ex_/542 ± 13.5 nm _Em_) was captured every 10s [36]. The related parameters for the CFP-YFP FRET pair were exactly the same as before [51], ECFP△C11 and mNG△N5 and mTurquoise2 and mNG△N5-mediated FRET were recalibrated and calculated as FRET_c_ = FRET_raw_ − F_d_/D_d_ × F_ECFP△C11_ − F_a_/D_a_ × F_mNG△N5_. In this formula, F_d_/D_d_ represents the measured bleed-through of ECFP△C11 into the FRET filter (0.73), and F_a_/D_a_ represents the measured bleed-through of mNG△N5 through the FRET filter (0.36). For mTurquoise2-mNG△N5 FRET measurements, bleed-through values were 0.25 for mNG△N5 and 0.65 for mTurquoise2. Normalized FRET was obtained by normalizing FRET_c_ against F_ECFP△C11_ or F_mTurquoise2_ to avoid differences in expression levels [51]. Fluorescence readings from regions of interest were exported from the SlideBook6.0.23 software and processed with Matlab 2023b to calculate the system-independent apparent FRET efficiency, FRET_c_/F_ECFP△C11_ or FRETc/F_mTurquoise2_. Representative traces from at least three independent experiments performed on 25–40 cells were shown as mean ± SEM.

In situ or in cellulo Ca^2+^ titration of STIM was performed using HeLa SK cells. For in cellulo measurements, cells transiently co-expressing YFP-SOAR1L with either PM-SC1111-CFP or PM-SC2211-CFP. The FRET signals of cells bathed in Ca^2+^ imaging solutions containing different concentrations of free Ca^2+^ were collected to obtain dose–response curves. Similarly, in situ measurements were conducted in cells transiently co-expressing mNG△N5-SOAR1L with either STIM1_1–310_-ECFP△C11 (SC1111-ECFP△C11) or variants. These measurements utilized a solution containing 10 mM NaCl, 140 mM KCl, 1 mM MgCl_2_, 20 mM HEPES, 0.025 mM digitonin, 0.01 mM ionomycin, and 1 mM EGTA (pH 7.4). During measurements, cells were permeabilized with the above solution containing different free Ca^2+^ concentrations, ranging from zero to 2 mM Ca^2+^ or up to 8 mM, to obtain corresponding Ca^2+^ responses. Ca^2+^ affinities of the STIM fragments or variants were then calculated by fitting the FRET-Ca^2+^ relationship to the Hill equation using Prism 9.5.1 software. All experiments were carried out at room temperature. Traces shown were representative of at least three independent repeats, with 30–60 single cells analyzed per repeat.

### 3.7. Confocal Microscopy

Images were taken using a ZEISS LSM880 system equipped with 63 × oil objective (NA 1.4) and controlled by ZEN 2.1 software. CFP or mTurquoise2, YFP or mNeonGreen, and mScarlet were excited by 405, 488, and 543 nm laser, respectively, and detected at 420–500 nm, 470–540 nm, and 590–690 nm. The thickness of the optical slice is 1 μm. The acquired images were analyzed using Image J 1.54f software (NIH) [36]. All experiments were repeated at least three times, and the representative data were shown.

### 3.8. Western Blotting

Total proteins were extracted using the Total Protein Extraction Kit (BB18011; BestBio, Beijing, China.) following the manufacturer’s instructions, and their concentration was measured by the BCA protein assay kit (E162-01; GenStar Biosolutions, Beijing, China). Lysates were prepared in nonreducing sample buffer containing 10% glycerol, 2% SDS, 65 mM Tris, and 0.005 mg/mL bromophenol blue and separated via SDS-PAGE either in the presence (reducing conditions) or absence (nonreducing conditions) of 50 mM DTT, followed by transferring to PVDF membranes (Millipore, Burlington, MA, USA). The resulting membranes were blocked for 1 h at 37 °C with TBST buffer (12 mM Tris-HCl, pH 7.5, 137 mM NaCl, 2.68 mM KCl, 0.1% Tween 20) containing 5% nonfat dried milk, then incubated with primary antibody overnight at 4 °C. After washing three times (10 min each) in TBST buffer, the membranes were loaded with secondary antibody for 40 min at room temperature. Detection was performed by ECL (GS009-4; Millipore) solution and imaged using the Tanon5200 detection system finally. HRP-labeled Streptavidin, STIM1, PDIA3, P4HB, and TXNDC5 were detected with anti-HRP-labeled Streptavidin (N100, Thermo Fisher scientific, Waltham, MA, USA), anti-STIM1 antibody (5668S; CST, Danvers, MA, USA) (1:1000 dilution), anti-PDIA3 antibody (159678-1-AP; Proteintech, Rosemont, IL, USA) (1:2000 dilution), anti-P4HB antibody (11245-1-AP; Proteintech, Rosemont, IL, USA) (1:1000 dilution), and anti-TXNDC5 antibody (19834-1-AP; Proteintech) (1:5000 dilution) followed by anti-rabbit-IgG (7074S; CST) (1:4000 dilution) respectively. Internal control β-actin was detected with anti-β-actin antibody (CW0096; CWBIO, Beijing, China) (1:4000 dilution), and the corresponding secondary antibody is anti-mouse-IgG (7076S; CST) (1:5000 dilution). The representative data shown were from three independent experiments. The intensity of the images was quantified by Image J 1.54f software, and the resulting data were plotted with Prism 9.5.1 software.

### 3.9. Statistical Analysis

All quantitative data are presented as means ± SEM of at least three independent biological repeats. Analysis of statistical significance was performed using unpaired Student’s *t*-test and paired Student’s *t*-test with GraphPad Prism 9.5.1 software, with a *p*-value < 0.05 considered statistically significant.

## 4. Conclusions

We identified P4HB and PDIA3, two luminal oxidoreductases within the ER dynamically associating with STIM1. This association potentially orchestrates redox modifications on STIM1’s two conserved cysteine residues (STIM1-C49-C56). Consequently, these two PDIs may fine-tune STIM1’s sensitivity to Ca^2+^ ions, thereby regulating its responsiveness to physiological cues and the subsequent generation of SOCE. This intricate mechanism likely plays a pivotal role in coordinating intracellular Ca^2+^ signaling and redox responses. Further exploration will undoubtedly shed light on the precise mechanisms by which these oxidoreductases influence intracellular Ca^2+^ signaling, as well as their specific roles in cell physiology and pathology.

## Figures and Tables

**Table 1:** Sequences of primers used for qPCR.

Target	Primers (5′→3′)	Sequences
PDIA3	Forward	CAAGCAGCGGGTTAGTGGT
	Reverse	TCTCGAAGTTGTCGTCCGTG
P4HB	Forward	TGCCAAGCAGTTTTTGCAGG
	Reverse	AATCTTCGGGGCTGTCTGCT
TXNDC5	Forward	GACATGTTCACGCACGGGAT
	Reverse	GGCTTGAAAAGCTTTAAGGTGGG
GAPDH	Forward	AACTGCTTAGCACCCCTGGC
	Reverse	ATGACCTTGCCCACAGCCTT

## Data Availability

The datasets generated during the current study are available from the corresponding author upon reasonable request.

## Supplementary Materials

The following supporting information can be downloaded at: https://www.mdpi.com/article/10.3390/ijms25147578/s1.
