# Supplementary material for: Redox Enzymes P4HB and PDIA3 Interact with STIM1 to Fine-Tune Its Calcium Sensitivity and Activation
Source: Int J Mol Sci. 2024 Jul 10;25(14):7578. doi: 10.3390/ijms25147578 (PMC11276767; doi:10.3390/ijms25147578)
Supplement: Supplementary file 1 [file ijms-25-07578-s001.zip › ijms-3045502-supplementary.pdf]

Figure S1

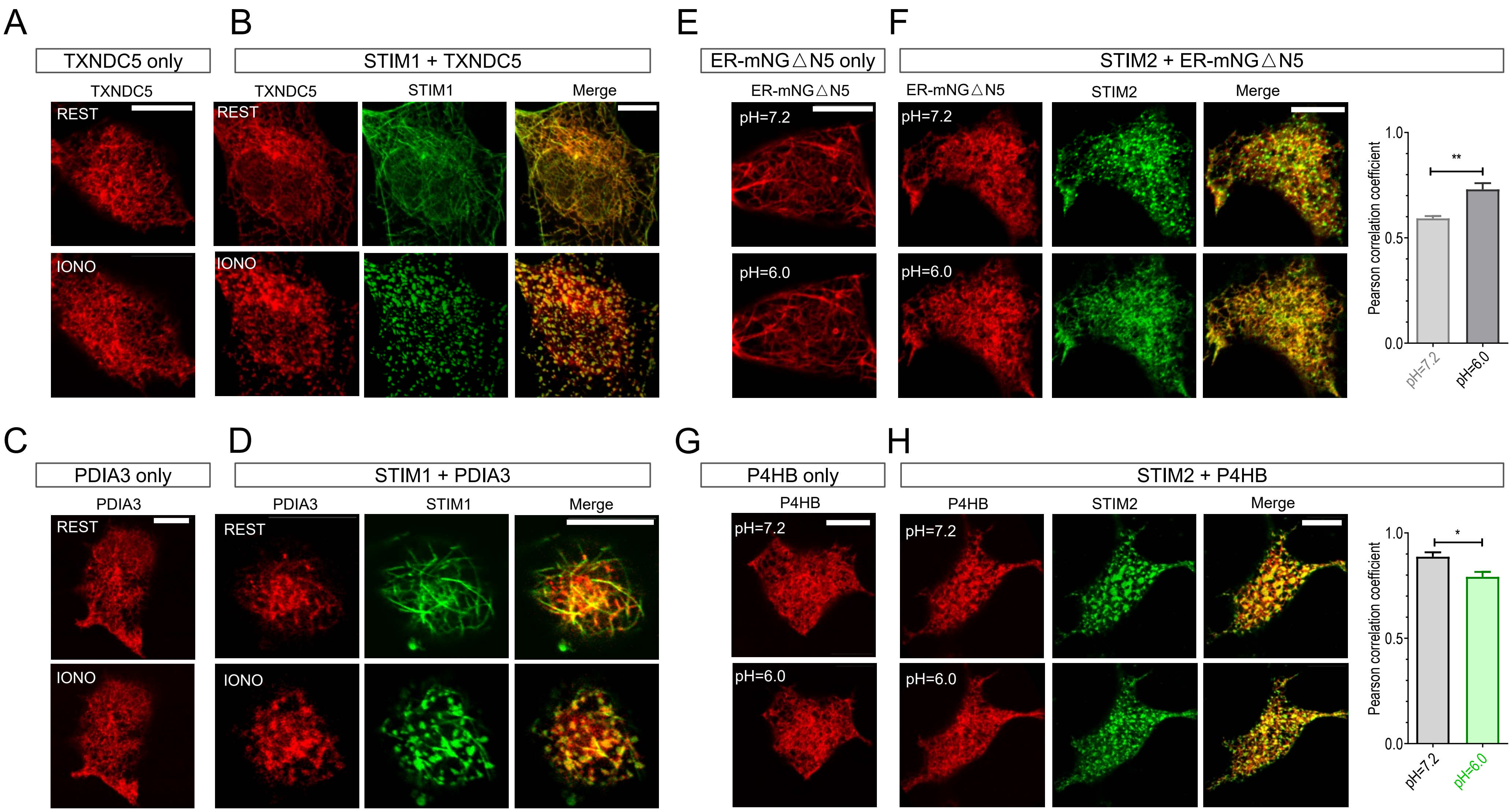

Figure S1. TXNDC5, PDIA3, or P4HB dynamically co-localized with STIM1 or STIM2 in HEK293 cells. HEK293 cells transiently ER-mNG△N5, TXNDC5-mScarlet, PDIA3-mScarlet, or P4HB-mScarlet either alone or together with mTurquoise2-STIM1 or mTurquoise2-STIM2 were examined with Airyscan super-resolution confocal imaging. Images of cells at basal condition and after 5-min ER-Ca^{2+}-store depletion with 2.5 µM IONO or intracellular acidification by 10 µM nigericin were collected from the same view field. Typical cellular images from three independent experiments were shown (more than 6 cells examined each time). STIM1 or STIM2 are shown in green, ER-mNG△N5, TXNDC5-mScarlet, P4HB-mScarlet, or PDIA3-mScarlet are shown in red. Scale bar, 10 µm. (**A**) TXNDC5 alone. (**B**) TXNDC5 co-expressed with STIM1. (**C**) PDIA3 alone. (**D**) PDIA3 co-expressed with STIM1. (**E**) ER-mNG△N5 alone. (**F**) ER-mNG△N5 co-expressed with STIM2. Bar chart on the right, statistics showing the extent of co-localization between STIM2 and ER-mNG△N5. (**G**) P4HB alone. (**H**) P4HB co-expressed with STIM2. The bar chart on the right displays the calculated Pearson-coefficient, serving as a quantitative measure of co-localization between STIM2 and P4HB. (n = 3, more than 18 cells examined in each group, *, $P < 0.01$; **, $P < 0.001$, Student's *t*-test).

Figure S2

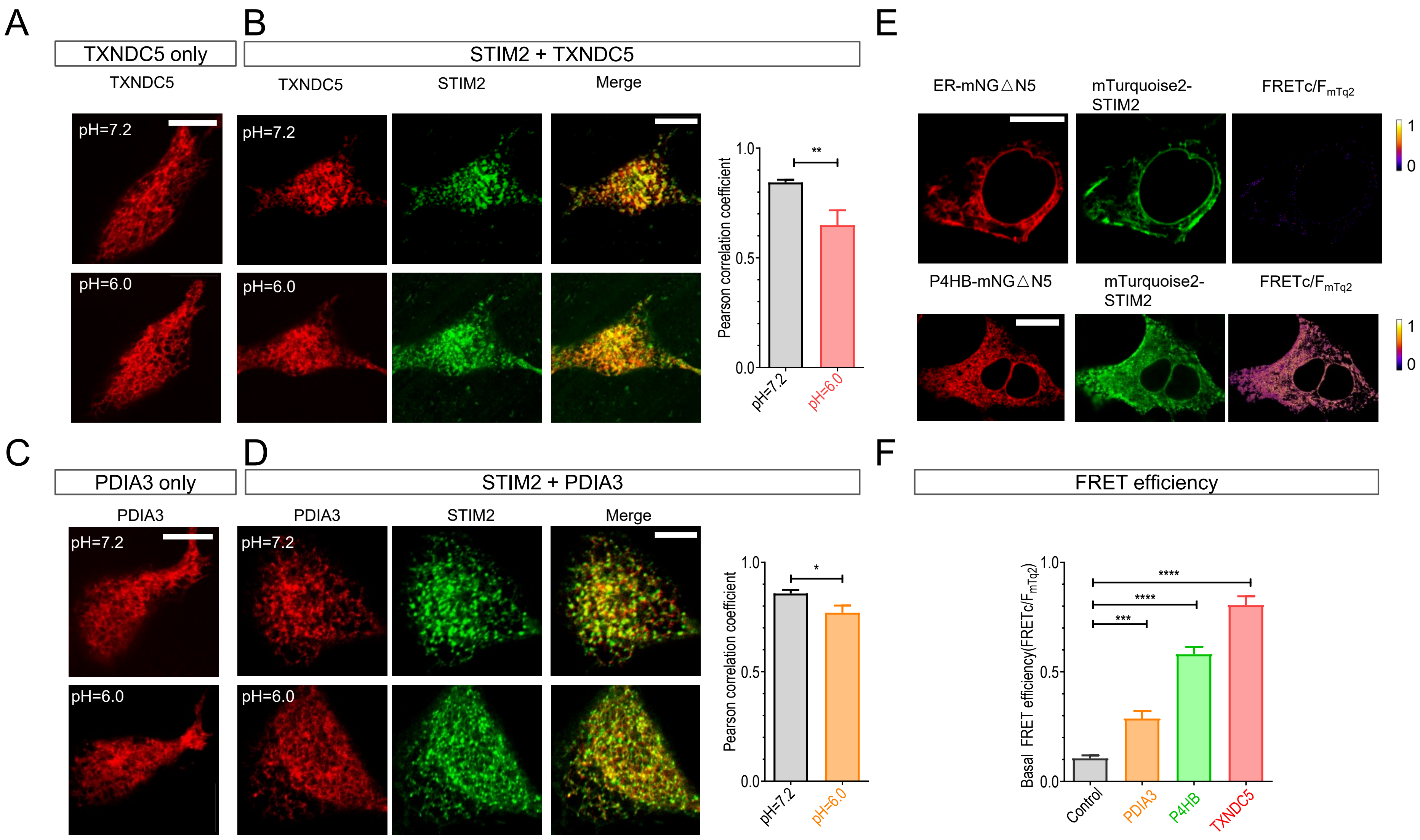

Figure S2. Colocalization or FRET signals between TXNDC5, PDIA3, or P4HB with STIM2 in HEK293 cells. HEK293 cells transiently TXNDC5-mScarlet, or PDIA3-mScarlet alone, or co-expressing mTurquoise2-STIM2 and TXNDC5-mScarlet, PDIA3-mScarlet, or P4HB-mNG△N5 were examined with Airyscan super-resolution confocal imaging. Images of cells at basal condition and after 5-min ER-Ca^{2+}-store depletion with 2.5 μM IONO or intracellular acidification by 10 μM nigericin were collected from the same view field. Typical cellular images from three independent experiments were shown (more than 6 cells examined each time). STIM2 are shown in green, TXNDC5-mScarlet, PDIA3-mScarlet or P4HB-mNG△N5 are shown in red. Scale bar, 10 μm. (**A**) TXNDC5 alone. (**B**) TXNDC5 co-expressed with STIM2. Bar chart on the right, statistics showing the extent of co-localization between STIM2 and TXNDC5. (n = 3, more than 18 cells examined in each group, **, $P < 0.001$, Student's *t*-test). (**C**) PDIA3 alone. (**D**) PDIA3 co-expressed with STIM2. The bar chart on the right displays the calculated Pearson-coefficient, serving as a quantitative measure of co-localization between the PDIA3 and STIM2. (n = 3, more than 18 cells examined in each group, *, $P < 0.01$, Student's *t*-test). (**E**) Representative cells showing the fluorescence (left two panels) or FRET efficiency (images on the right) of cells co-expressing mTurquoise2-STIM2 and either ER-mNG△N5 (top) or P4HB-mNG△N5 (bottom). (n = 3, more than 20 cells examined each time). (**F**) Statistics of showing basal FRET efficiency between mTurquoise2-STIM2 and ER-mNG△N5, PDIA3-mNG△N5, TXNDC5-mNG△N5 or P4HB-mNG△N5. (n = 3, more than 20 cells examined each time, ***, $P < 0.007$; ****, $P < 0.0001$, unpaired Student's *t*-test).

Figure S3

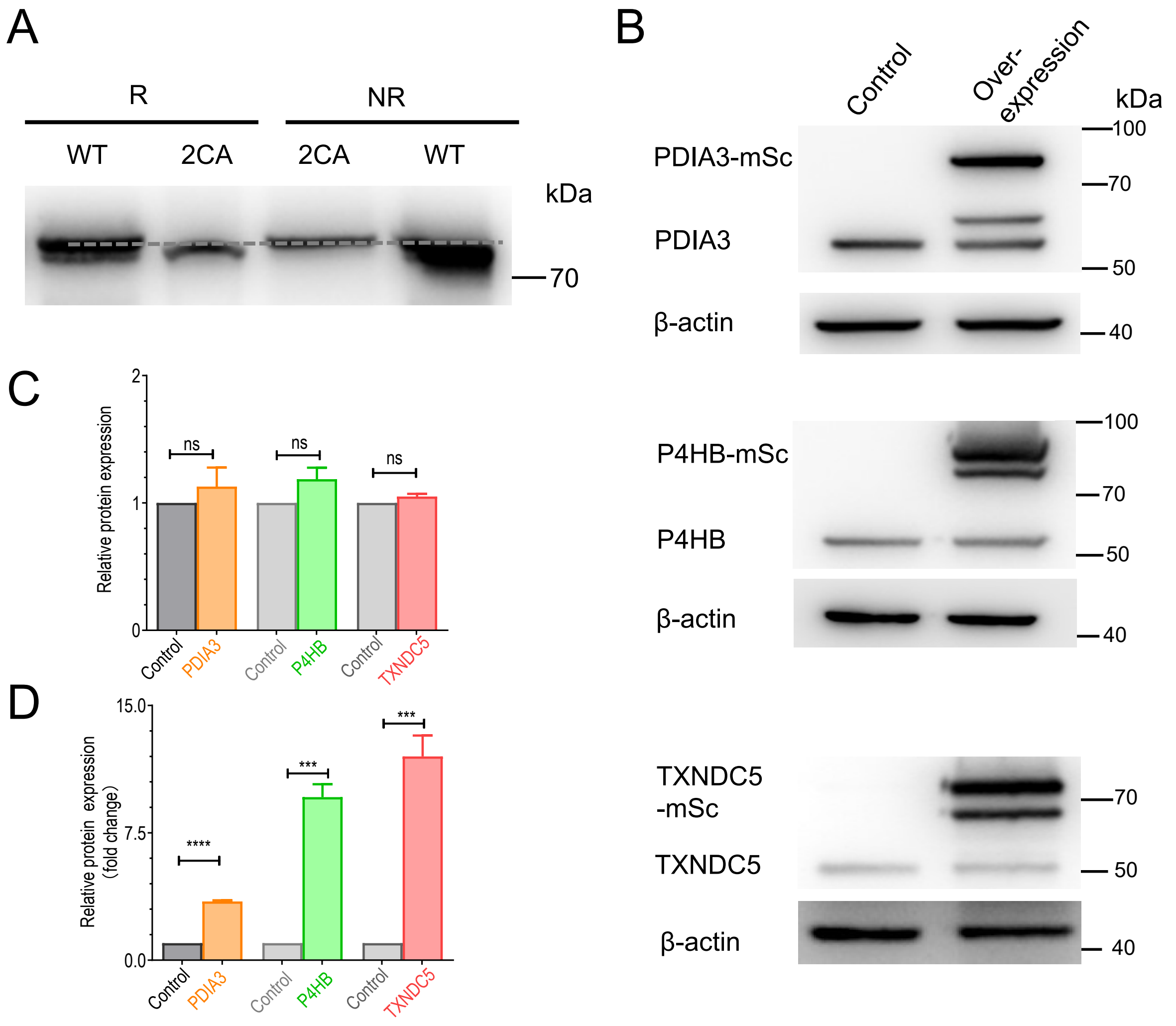

Figure S3. Immunoblot analysis of STIM1$_{1\text{-}310}$-ECFP△C11, PDIA3, P4HB, or TXNDC5 protein levels. **(A)** Immunoblot analysis of STIM1$_{1\text{-}310}$-ECFP△C11 under reducing (R) and non-reducing (NR) conditions. Lysates from HeLa-SK cells expressing STIM1$_{1\text{-}310}$-ECFP△C11 (WT) or STIM1$_{1\text{-}310}$-2CA-ECFP△C11 (2CA) were separated via SDS-PAGE under reducing and non-reducing conditions. The dashed line indicates the position of the STIM1-1-310-ECFP△C11 molecular weight marker (kDa) under reducing conditions. (n=3). (**B**) Typical Western blot images. Lysates from cells overexpressing PDIA3-mScarlet, P4HB-mScarlet, TXNDC5-mScarlet or a blank control plasmid were used for the analysis. β-actin was used as an internal loading control. (**C**) Statistics showing the effects of overexpression on the levels of respective endogenous proteins. The levels of each protein was normalized to the corresponding β-actin signal with Image J. (**D**) Statistics showing the total levels of each target protein in overexpressing cells normalized to the corresponding endogenous protein in blank control cells. (***, $P < 0.0003$; ****, $P < 0.0001$; ns, not significant, Student's *t*-test, n= 3).

Figure S4

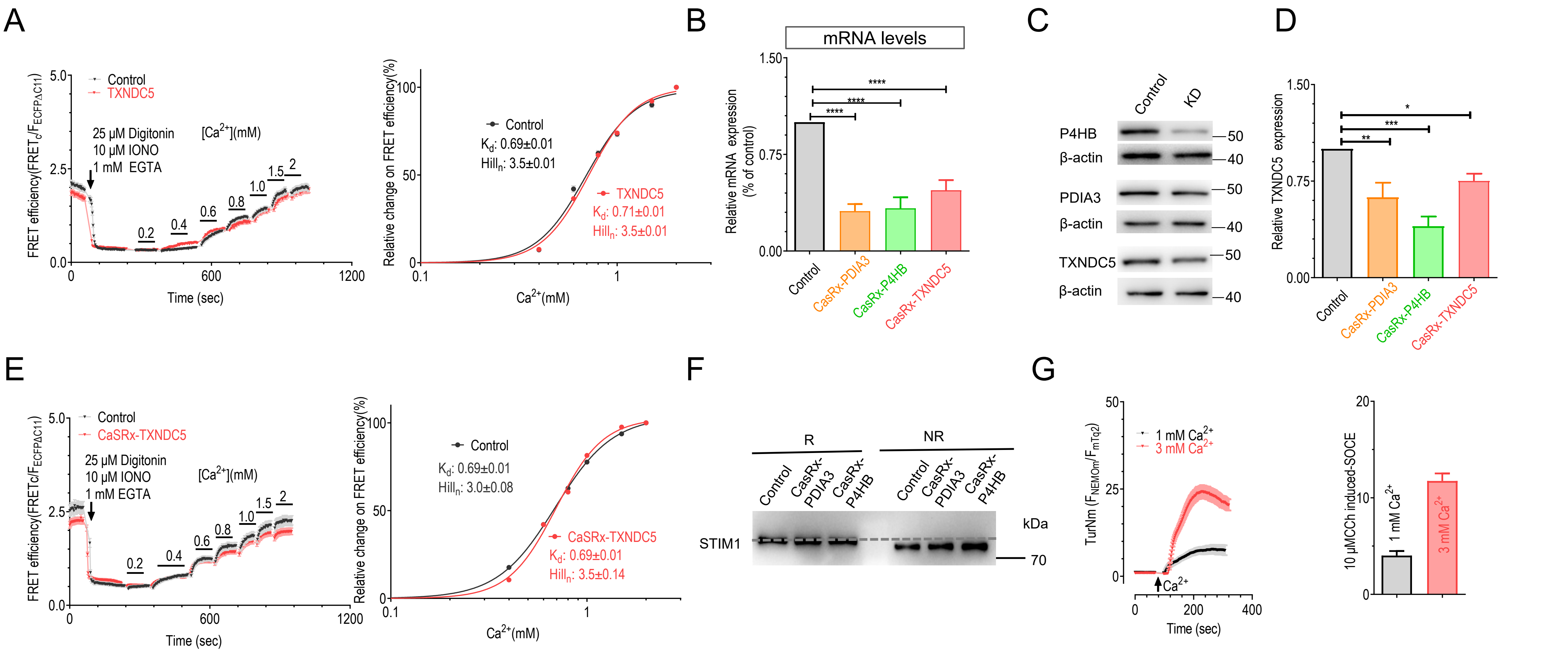

Figure S4. Overexpressing or knocking down TXNDC5 did not affect the Ca^{2+}-binding affinity of STIM1 in HeLa-SK cells. (A) *In situ* Ca^{2+}-titration responses of mNG△N5-SOAR1L and $STIM1_{1-310}$-CFP△C11 co-expressed in HeLa-SK cells transfected with TXNDC5-mScarlet or mScarlet. Left, traces of Ca^{2+} response; right, Ca^{2+}-titration curves. (n = 3, more than 30 cells examined each time). This assay was performed together with those shown in Fig. 3A, thus the same control was used. **(B)** Statistics showing relative mRNA levels of PDIA3, P4HB or TXNDC5 in cells transfected with CasRx empty vector or corresponding sgRNAs. (n = 3, ****, P < 0.0001, Student's t-test). (**C**) Immunoblot analysis of P4HB, PDIA3, or TXNDC5 protein levels in cells transfected with CasRx empty vector , CasRx-P4HB, CasRx-PDIA3, or CasRx-TXNDC5, and β-actin was used as loading control. (**D**) Quantification of the identified proteins was conducted for the Western blotting shown in (D). (n = 3, *, P < 0.01; **, P < 0.004; ***, P < 0.0002, Student's t-test). (**E**) *In situ* Ca^{2+}-titration curves shown by FRET signals between mNG△N5-SOAR1L and $STIM1_{1-310}$-CFP△C11 constructs in HeLa-SK cells transfected with CasRx empty vector or TXNDC5 sgRNA. Left, traces of FRET responses; right, Ca^{2+}-titration curves. This assay was performed together with those shown in Fig. 3B, thus the same control was used. (n = 3, more than 30 cells examined each time, ns, not significant, unpaired Student's t-test). (**F**) Immunoblot analysis of STIM1 under reducing (R) and non-reducing (NR) conditions. Lysates from HeLa cells transfected with CasRx empty vector, CasRx-P4HB, or CasRx-PDIA3 were separated via SDS-PAGE under reducing and non-reducing conditions. The dashed line indicates the position of the STIM1 molecular weight marker (kDa) under reducing conditions. (n = 3). (**G**) Comparison SOCE responses of TurNm cells bathed in extracellular solutions containing different concentration of Ca^{2+}. Prior to recordings, cell were incubated in nominally Ca^{2+} free solutions containing 10 μM CCh for 5 min. 10 μM CCh was present throughout recordings. Left, typical traces; right, statistics. (n = 3).
